# Early childhood deprivation and the impact of negative life events on mental health in later life: a test of the stress sensitization hypothesis

**DOI:** 10.3389/fpsyt.2024.1393107

**Published:** 2025-01-07

**Authors:** Jala Rizeq, Mark Kennedy, Kirellos Miseih, Wangjingyi Liao, Edmund J. S. Sonuga-Barke

**Affiliations:** ^1^ School of Health and Wellbeing, University of Glasgow, Glasgow, United Kingdom; ^2^ Department of Child and Adolescent Psychiatry, Institute of Psychiatry, Psychology and Neuroscience, King’s College London, London, United Kingdom

**Keywords:** deprivation, stress sensitization, mental health, adoptees, life event

## Abstract

**Introduction:**

Early life exposure to adversity and stress has been shown to sensitize young people to later negative life events (LEs), leading to increased susceptibility to mental health problems. We explored this question by testing whether exposure to severe institutional deprivation moderated the effect of adolescent exposure to LE on early adult depression and anxiety. To test the specificity of these effects, we contrasted the effects on these outcomes with neuro-developmental problems (autism and disinhibited social engagement), known from previous studies to be associated with deprivation from early childhood.

**Methods:**

Participants were 170 adoptees from the English and Romanian Adoptees study. Of these, 124 (66 females) grew up as infants and small children in severely depriving Romanian orphanages before being adopted into UK families before the age of 43 months. The remainder were UK adoptees (16 females) with no history of deprivation who were placed before the age of 6 months. For this analysis, data on emotional problems, autism, and disinhibited social engagement were used at both age 15 years and in early adulthood (23–25 years) using standardized questionnaire and interview measures. Exposure to independent, dependent, and peer-related LE was measured at age 15 years.

**Results:**

In all models, there were continuities in all outcomes between adolescence and adulthood (*p*s < .05). Dependent LE had a main effect on emotional symptoms, with higher exposure to dependent LE predicting an increase in emotional symptoms between age 15 and young adulthood. For independent and dependent LE, there were no interactions between deprivation and LE. For peer-related LE, the interaction was significant for emotional problems, but not deprivation-specific problems (i.e., autism/disinhibited social engagement)—the group of individuals exposed to early extreme deprivation and elevated peer-related LE had elevated emotional problems.

**Discussion:**

There was no evidence that early severe institutional deprivation increased susceptibility to depression and anxiety following exposure to independent or dependent LE in general. However, it appeared to sensitize adolescents to the effects of peer-related LE specifically. We discuss possible mechanisms by which difficulties in peer relations might influence the emergence of depression and anxiety to already vulnerable individuals.

## Introduction

Individuals exposed to severe institutional deprivation in early childhood are at increased risk of a range of neurodevelopmental and mental health conditions (1). These can persist even after affected individuals have left institutional and entered adoptive or foster care [e.g., (2–8)]. For example, in the English and Romanian Adoptees (ERAs) Study, children were exposed to up to 43 months of severe deprivation in Romanian orphanages in the 1980s before being adopted into UK families. Follow-up studies of this group found that those exposed to periods of deprivation lasting longer than 6 months were at a substantially elevated risk of neurodevelopmental conditions (6), including consistently elevated symptoms of attention-deficit/hyperactivity disorder (ADHD; 4), autism (9), and disinhibited social engagement from childhood through young adulthood (4). Such strong persistence of neurodevelopmental problems despite environmental enrichment following adoption is consistent with the hypothesis that exposure to deprivation during critical and sensitive developmental periods can create long-term risk by bringing about alterations in brain structure and function (10). This hypothesis is supported by longitudinal brain imaging studies showing marked effects on brain function and structure many years after deprivation exposure ended [e.g., (11–13)].

Interestingly, even in those exposed to extended deprivation, emotional problems were notable by their relative absence in childhood and early adolescence (14). However, a precipitous increase in depression and anxiety symptoms occurred in their transition from adolescence to adulthood (6). This suggests that while the primary effects are neurodevelopmental, institutional deprivation has secondary effects on broader mental health. Golm et al. (2) explored why this was the case and found that this mental health vulnerability was mediated, first, by early developing deprivation-related neurodevelopmental problems and secondarily by unemployment and friendship difficulties in young adulthood. In this paper, we test an alternative mechanism by which early institutional deprivation might impact later mental health. We test deprivation’s role in creating susceptibility to the effects of post-institutional environmental exposures to negative LE. In general terms, negative LE experienced in childhood and adolescence increases the risk of mental health problems [e.g., (15, 16)]. However, individuals differ in the extent to which this is the case—some are more susceptible and some less. Such susceptibility can be explained by both genetic and environmental exposures (see 17). While most studies have focused on understanding the genetic roots of susceptibility (18), in the current study, we focus on the environmental origins of susceptibility to the negative effects of LEs, in this case, institutional deprivation. In this, we are guided by the stress sensitization hypothesis, rooted in diathesis-stress models of depression. This posits that exposure to early adversity during critical periods programs the stress neurobiology to sensitize an individual to the effects of later stressful LEs, making them more susceptible to its negative effects (19). This effect has been shown in the context of emotional and somatic psychopathology, whereby early adversity sensitized individuals to the effect of later stress on mental health outcomes in adolescents and adults (19–22). Further, McLaughlin et al. (23) reported stress sensitization effects for depression, posttraumatic stress disorder, and other anxiety disorders in a population-based sample of adults.

The stress sensitization effect may vary as a function of the type of stressful life event experienced, especially in relation to their controllability. Independent LEs are those which an individual cannot control (e.g., loss of a loved one), while dependent LEs are those that an individual can control (e.g., relationship difficulties; 24). Some research has shown that it is dependent events, not independent events, that elevate mental health risk in youth (25, 26). Furthermore, in a sample of young adults, the stress sensitization effect on depressive symptoms was observed only for dependent events (27). Further, Gao et al., found that early maltreatment sensitized adolescents to dependent events in relation to non-suicidal self-injury (28). Other research has shown the obverse: stress sensitization reported in response to independent and not dependent events. Wade et al. (29) found that independent stressful LEs at age 12 years predicted changes in externalizing problems at age 16 years in the group with history of institutionalization (i.e., early adversity) but not in the group without such history. They did not find significant effects of dependent stressful LEs. Peer-related LEs are distinguished from independent and dependent LEs, because they may have both dependent and independent characteristics (30). For instance, victimization in adolescence, which may or may not be under the control of the victim, are potent predictors of mental health outcome in later life [see review (31)], and as such, it is important to understand whether this effect is affected by pre-existing susceptibility.

In the current study, we extend this research by asking the question: Does early exposure to institutional deprivation sensitize individuals to the effect of negative LE, making individuals more susceptible to mental health problems? More specifically, we interrogate data from the ERA study to test whether the effects of adolescent negative LE on the emotional problems of adoptees are moderated by their levels of pre-exposure to extreme institutional deprivation. We will contrast these effects for emotional problems with those for adult autism and disinhibited social engagement, shown in previous studies to be specific sequelae of institutional deprivation of early onset [deprivation-specific problems—DSPs; (32, 33)]. We hypothesize that these outcomes would not be affected by exposure to adolescent LE, irrespective of exposure to prior institutional deprivation. We will explore differences in these effects for independent, dependent, and peer-related LE.

## Methods

### Participants

The ERA study included 165 Romanian (between 1 and 43 months in institutions; 91 females) and 52 UK adoptees (adopted before the age of 6 months with no deprivation history; 18 females) and their adoptive families who entered the ERA study in the mid-1990s [for more details, see (6)]. Data for this study were constrained to a total of 170 participants who had available life event data at age 15 years. Of those, 46 were UK (16 females) and 124 were Romanian (66 females) adoptees.

### Procedure and measures

Data utilized for this study were taken from assessments carried out at age 15 years and in young adulthood (22–25 years). The procedure is described in detail in previous work utilizing these data [see (6)]. Primary assessments took place in the individuals’ homes. Some questionnaire measures at the young adult follow-up were completed online or returned by post. For practical and scientific reasons, different assessment instruments, including interviews and standardized questionnaires, were used at different ages. Primary outcome variables in young adulthood used in the current analysis included emotional symptoms and deprivation-specific problems (i.e., a composite of ASD and DSE symptoms). The main predictor variable was institutional deprivation, and the main moderator included age 15 years’ self-ratings of exposure to LEs, specifically dependent LE, independent LE, and pee-related LE.

### Variables

#### Institutional deprivation

As in previous analyses (6), we divided the Romanian adoptees into two groups to capture the effect of deprivation duration in the ERA—those who spent more than 6 months in institutions (high deprivation group) and those who spent less (low deprivation group). Previous analyses have shown a step change, at this value, in cognitive, neurodevelopmental, and mental health outcomes across all ages. Romanian adoptees who experienced less than 6 months of deprivation were largely unaffected by their experience, with outcomes in the normal range and no difference on average compared with the non-deprived controls, while those over 6 months showed significant and substantial impairment across a broad range of outcomes, including clinically significant symptoms of ADHD, ASD, and DSE (2, 4, 6, 14). However, the rates of disorder and impairment within this group did not increase further with additional months in the institution after the 6-month point. As in previous analyses, we then combined the <6-month group with the UK adoptees, with which they did not differ in terms of outcomes, to create a larger low deprivation group (low deprivation group *n* = 100 and high deprivation group *n* = 70).

#### Life event exposure

We assessed independent LE (17 items; Cronbach’s alpha = .55), dependent LE (6 items; Cronbach’s alpha = .56), and peer-related LE (4 items; Cronbach’s alpha = .78) using self-report at age 15 years including an amended version of the of LE Questionnaire (34). The full list of items is included in the **Appendix**.

#### Deprivation-specific problems

Composite scores of ASD and DSE z-scores were used at age 15 years and young adulthood to represent DSPs. Parent ratings on a 15-item version of the Social Communication Questionnaire (6, 35), adapted to be appropriate in adolescence and adulthood, were used to index of autism symptoms at age 15 years (Cronbach’s alpha = .82) and young adulthood (Cronbach’s alpha = .88). Five items were sampled from each scale—social reciprocal interaction, communication, and repetitive and stereotyped behaviors (see 6 for the item selection rationale). Items were rated as absent (0) or present (1). DSE symptoms were measured based on researcher ratings of parents’ responses to age-appropriate variations of three interview questions at age 15 years (Cronbach’s alpha = .86) and young adulthood (Cronbach’s alpha = .71). The questions were in relation to interactions with strangers, tapping the constructs of being “too friendly,” showing “inappropriate intrusiveness,” and being “unaware of social boundaries” (6). A rating of “definite evidence of disinhibition” (rating of 2 on a 0–2 scale) represented a positive endorsement.

#### Internalizing symptoms

In adolescence, emotional problems/internalizing symptoms were based on the Strengths and Difficulties Questionnaire [SDQ; (36)], parent report. Specifically, endorsements on items 8; Many worries, often seems worried, 13. Often unhappy, downhearted or tearful and 16. Nervous or clingy in new situations, easily loses confidence were summed to create a total score [see (6)], with Cronbach’s alpha = .60. The three items were used and validated in the 2017 paper to enable assessment of change over time using a consistent list of items used across timepoints. In this paper, we used the three-item measure for age 15 years to control for its effect on the young adult internalizing outcome score using a longer version of the measure.

In adulthood, emotional problems/internalizing symptoms were based on symptom counts of generalized anxiety disorder (GAD) and major depressive disorder (MDD) based on DSM-5 criteria (37), scored via the Conners Comprehensive Behavior Rating Scales [CBRS; (38)] parent-report, with Cronbach’s alpha = .63. Parent reports were chosen on the basis of consistency with measures taken at age 15 years, strong associations between self-report and parent report, more parent-report data being available, and to reduce shared method variance (2).

### Data analysis

All analyses were conducted using *Rstudio* version 2022.07.1. Descriptive statistics and bivariate correlations were generated for all variables. Moderated regression models were used to test the moderating effect of deprivation exposure (<6 and >6 months) on the effect of age 15 years stressful LE on young adulthood outcomes while controlling for the effect of the outcomes at age 15 years. Separate regression models were estimated for the three domains of stressful LE (i.e., independent, dependent, and peer-related stressful LE). The QuantPsyc package was used to obtain standardized regression coefficients.

## Results

### Descriptive statistics and correlations

**Table 1** presents the descriptive statistics for all variables. Correlations among variables are reported in **Table 2**. Duration of deprivation was positively and significantly associated with all variables except for dependent LEs. All three domains of events were positively and significantly correlated with each other and with emotional symptoms and deprivation-specific problems (autism and DSE composite score) in adolescence and young adulthood. Independent and peer-related events were positively and significantly correlated with emotional symptoms and deprivation-specific problems at age 15 years, whereas dependent stressful LE was only positively and significantly associated with deprivation-specific problems at this age.

**Table 1:** Descriptive statistics.

Variable	*n*	Mean	SD	Range
Adolescent exposures				
Dependent EE	169	1.30	1.19	0, 5
Independent LE	168	2.68	1.97	0, 10
Peer-related LE	170	0.82	1.25	0, 4
Adolescent outcomes				
Emo symptoms	159	0.17	0.51	0, 3
DSP	157	0.00	0.83	−0.42, 3.22
Young adulthood outcomes				
Emo symptoms	128	3.74	4.4	0, 16
DSP	119	−0.06	0.74	−0.54, 2.07

**Table 2:** Correlations among variables.

Variable	1	2	3	4	5	6	7	8	9
1 .Sex	1								
2. Deprivation	−.15	1							
Adolescent exposures									
3. Peer-related LE	−.02	.21*	1						
4. Independent LE	.00	.19*	.38*	1					
5. Dependent LE	.10	−.05	.30*	.37*	1				
Adolescent outcomes									
6. Emo symptoms	−.10	.17*	.15	.17*	.09	1			
7. DSP	−.07	.38*	.31*	.33*	.20*	.24*	1		
Young adult outcomes									
8. Emo symptoms	−.12	.23*	.24*	.23*	.18*	.30*	.43*	1	
9. DSP	−.16	.37*	.31*	.23*	.22*	.22*	.70*	.41*	1

*p <.05; DSP, deprivation specific problems (DSE + autism); Emo, emotional; LE, life event; Deprivation, deprivation group.

### Deprivation x LE moderation analysis

**Table 3** shows the findings for all regression models.

**Table 3:** Regression models with three domains of LE.

	Young adult outcomes					
	Emotional symptoms			DSP		
	B	*B*	*P*-value	B	*B*	*P*-value
Independent LE						
Main effects model						
LE	0.35	.16	.07	−0.01	−.01	.84
Deprivation	1.27	.14	.11	0.17	.11	.15
Outcome at age 15	1.89*	.24	.01	0.58*	.65	<.001
Interaction effect model						
LE	0.32	.15	.23	0.01	.03	.74
Deprivation	1.08	.12	.43	0.28	.18	.14
Outcome at age 15	1.90*	.24	.01	0.59*	.66	<.001
LE × deprivation	0.07	.03	.86	−0.04	−.11	.46
Dependent LE						
Main effects model						
LE	0.81*	.21	.02	0.04	.06	.44
Deprivation	1.55*	.17	.047	0.18	.11	.13
Outcome at age 15	1.88*	.24	.01	0.56*	.63	<.001
Interaction effect model						
LE	0.36	.09	.46	.02	.03	.79
Deprivation	0.53	.06	.63	.13	.09	.39
Outcome at age 15	1.85*	.24	.01	.55*	.62	<.001
LE × deprivation	0.85	.22	.20	.04	.06	.67
Peer-related LE						
Main effects model						
LE	0.73*	.20	.02	0.05	.07	.34
Deprivation	1.11	.12	.17	0.16	.10	.17
Outcome at age 15	1.98*	.25	<.01	0.55*	.62	<.001
Interaction effect model						
LE	−0.15	−.04	.77	.04	.07	.54
Deprivation	−0.19	−.02	.84	.16	.10	.24
Outcome at age 15	2.32*	.30	<.001	.55*	.62	<.001
LE × deprivation	1.47*	.40	.03	.00	.01	.97

Outcome at age 15 years matches the outcome in the model. DSP, deprivation-specific problems (DSE + autism); LE, life event; Deprivation, deprivation group. *p <.05.

*Independent LE.* DSPs and emotional problems at age 15 years significantly predicted their equivalent outcome in young adulthood (*p*s <.05). The independent LE scores at age 15 years predicted neither young adult outcome (*p*s >.05). The level of deprivation was not associated with young adult outcomes after other factors were considered. The interaction between deprivation and adolescent-independent LE was not significant for either outcome.

*Dependent LE.* Adolescent DSP and emotional problems predicted their equivalent outcome in young adulthood. The interactions for either outcome between early deprivation and dependent LE at age 15 years was not significant. The main effects of dependent LE and level of deprivation were significant in the emotional symptoms model, whereby exposure to institutional deprivation for more than 6 months and higher levels of dependent LE in adolescence were uniquely associated with higher emotional symptoms in young adulthood, while controlling for emotional symptoms in adolescence.

**Figure 1:**
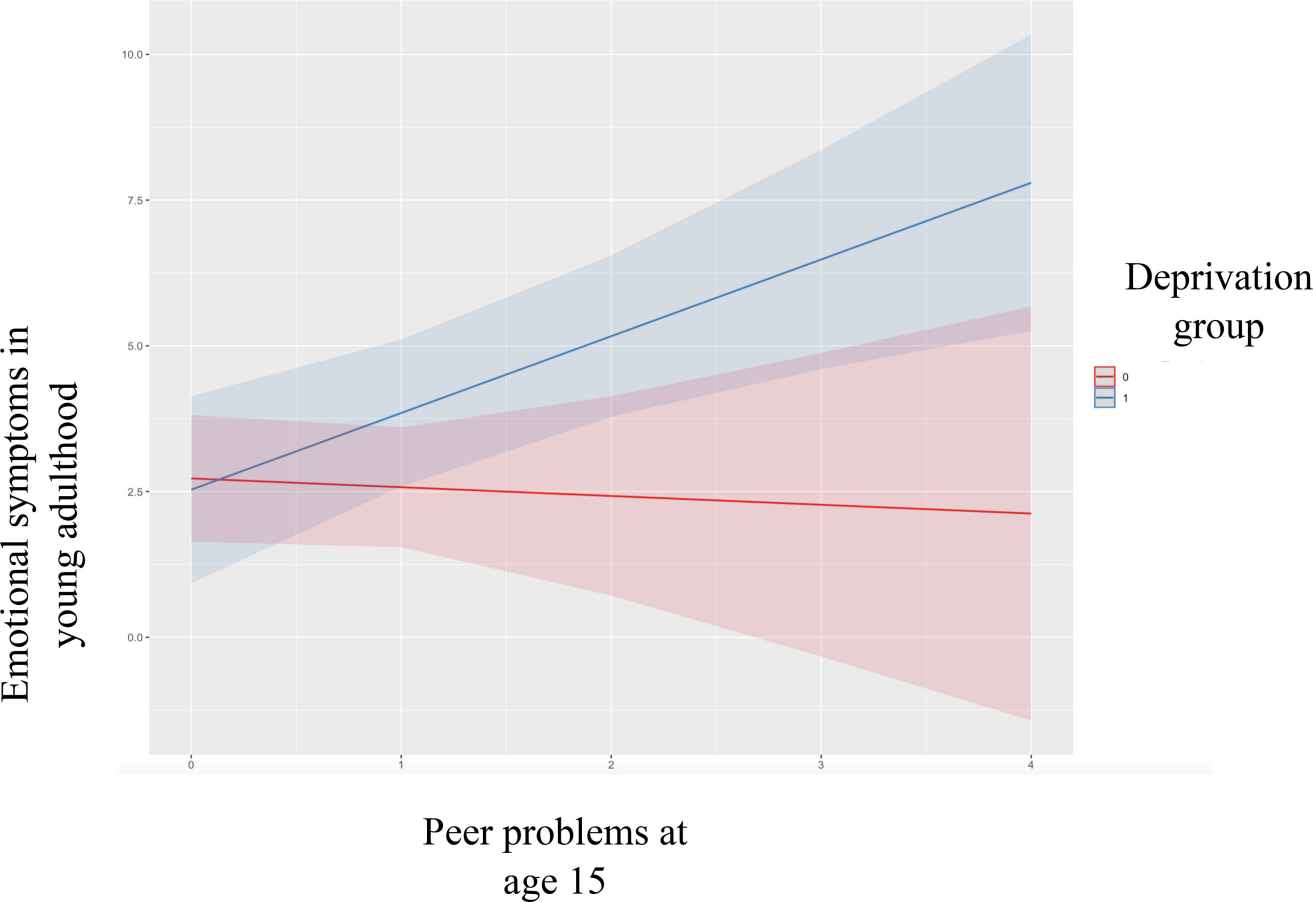
Interaction plot for Peer LE and Deprivation group on emotional symptoms. 0= Low Deprivation Group and 1= High Deprivation Group.

*Peer-related LE.* Adolescent DSP and emotional problems predicted their equivalent outcome in young adulthood. In the main effects model, adolescent peer LE was associated with emotional symptoms, but not DSP, in young adulthood. Deprivation did not predict either adult outcome once other factors were controlled. The interaction between peer LE and deprivation was significant in the emotional symptoms model (*p* = .03; see **Figure 1**) but not the DSP model. The interaction is plotted in **Figure 1**—increased peer-related LE only predicted more emotional problems in high-deprivation group.

## Discussion

In this study, we show limited evidence for a general stress sensitization effect of stressful LE following early institutional deprivation. The exception to this was peer-related LE, which appeared to be more closely related to poorer mental health problems in those exposed to early adversity versus those without such a background. Exposure to dependent stressful LE in adolescence predicted increases in emotional problems in young adulthood irrespective of level of deprivation.

There was no evidence of stress sensitization due to early deprivation experience in relation to exposure to independent or dependent negative LE. In examining emotional problems or internalizing psychopathology, previous research also did not find that early adversity sensitized individuals to the effects of independent LE [e.g., (27, 28)]. It is possible that the dependent LE queried in this sample were distinct from events measured elsewhere. For example, in studies by Gao et al. (28) and (27), their list of dependent LE included arguments and fights with peers or being hurt by a peer that would have been captured in the peer-related stressful LE in our study. Nonetheless, exposure to dependent stressful LE in adolescence still predicted an increase in emotional problems in young adulthood when controlling for level of early deprivation, sex, and level of emotional problems in adolescence. Therefore, it appears that the effect of this type of stressful LE consistently increased the risk for the emergence of later emotional problems, irrespective of level of early institutional deprivation. It is notable that exposure to dependent LE was the only domain that did not differ across deprivation groups. The findings are consistent with work showing that dependent stressful LE were associated with elevated depressive symptoms in adolescence regardless of the level of childhood adversity experienced (39). These findings follow from research that shows that dependent stressful LE generally has stronger associations with emotional problems than independent stressful LE [e.g., (40)]. According to Fassett-Carman et al. (40) study, this association could be further characterized by the subjective appraisal of uncontrollability of these types of events. Such that people who rate dependent stressful LE more highly on uncontrollability are more likely to experience higher emotional problems. Another important factor that can impact how one responds to interpersonal stress and, in turn, the level of emotional problems experienced is self-worth and self-esteem (41, 42).

In contrast, our findings provide evidence for the late emerging emotional problems in young adulthood being partly a function of early deprivation sensitizing individuals towards a greater impact of peer-related LEs in adolescence. This effect was specific to exposure to peer-related LE in adolescence. Peer problems and victimization are prevalent in adolescence and lead to later mental health difficulties [see (43)]. Even when it is not the most prevalent stressor, it has one of the strongest effects on emotional problems (15). Nonetheless, not everyone exposed to peer problems goes on to experience major difficulties. In this study, we show that this effect on emotional problems in young adulthood is moderated by the level of early deprivation. Specifically, those individuals exposed to extreme levels of deprivation due to institutionalization for more than 6 months were at increased risk of developing emotional problems in young adulthood as a function of peer-related LE in adolescence. This risk was not observed in the low deprivation group. This increased vulnerability to stress observed in the high deprivation group could be explained by the strong support for the dysregulation of the stress response system—hypothalamic-pituitary-adrenal (HPA) axis—whereby individuals with early-life adversity show blunted cortisol levels in response to acute social stress [see meta-analysis (44)]. It is also notable that exposure to peer-related LE is higher in the high deprivation group, which suggests that those with extreme early institutional deprivation are at risk of exposure to more peer-related stressful LE. In our previous work, we have shown that this vulnerability to peer problems and victimization is in part a function of DSPs (33). Taken together, peer-related processes appear to be heavily involved in mental health outcomes for children and adolescents with early extreme deprivation experiences.

Further, none of the domains of LE at age 15 years had primary effects on DSPs in young adulthood. There are several possible reasons for this (6, 33). Recent longitudinal research has shown that within the context of early deprivation, DSPs’ association with negative peer-related LE (i.e., victimization) across childhood and adolescence is one of stress generation, whereby symptoms of DSPs increase risk of exposure to peer victimization (33). This has been further supported in research showing that increased ADHD symptoms explained increases in social isolation rather than the other way around (45). This then speaks to DSPs being early emerging within the context of extreme deprivation but consistent in presentation over time.

Given that our findings appear to be primarily inter-personal, it is possible to view this through the lens of Bowlby's attachment theory (46). Due to the caregiving circumstances, children in the Romanian orphanages were not able to form selective attachments with staff members, but as Bowlby originally argued, siblings and others may play some role in shaping attachments or at least related internal working models (47). Unfortunately, no such data were gathered in the orphanages, and given the physical confines in those orphanages, it is unclear whether the necessary separation/reunion/facilitation of exploration experiences could have been had with any other children. Nevertheless, attachment-focused analyses show that, even at a very early age, it was evident that many of even the most deprived children had formed secure attachments with their adoptive parents (48). Subsequently, distinctions between those found to be securely attached versus insecure (or indeed those with selective vs. non-selective attachment patterns) were found on the whole to not predict poorer outcomes in later life [e.g., (49)]. Furthermore, an initial lack of selective attachments in the early years post-placement in adoptive homes may be due to underlying neurodevelopmental symptomtology rather than disruptions in attachment playing a causal role in the outcomes we see here (49).

Taken together, these results provide further mechanistic evidence explaining the late emergence of emotional problems in young adulthood in individuals who experienced extended periods of early institutional deprivation. In addition, we supported our hypothesis that the stress sensitization effect, when present, was specific to emotional problems in young adulthood and did not generalize to DSPs (autism and DSE).

### Strengths and limitations

This study builds on previous literature in several ways. First, it relies on documented levels of institutional deprivation rather than retrospective reports of early life adversity. Second, it uses multi-wave prospective data with long intervals that would allow us to capture developmental change. Further, controlling for levels of outcomes in adolescence ensures that we capture change in outcome that is unique to exposure and stress sensitization. In terms of limitations, we cannot distinguish different aspects of the original exposure, and thus it has been viewed as global deprivation, even though we cannot rule out abuse as a factor. In addition, our measures of post-adoption LEs are limited in a number of ways, as some had to be shortened to assist with longitudinal follow-ups and consistency across timepoints. This limits the psychometric properties of the tools. Statistical power was also limited by the initial sample size and by the sample size of those who completed LE questionnaires.

## Conclusion

In summary, our findings provide limited support for the effect of LEs (either dependent or independent) being sensitized by a history of institutional deprivation. Instead, our findings suggest that there may be a specific effect of sensitization towards social/peer-related LE. Even so, rather than a general effect, this impacted specifically on depression and anxiety and not the neurodevelopmental problems seen within the cohort. Alongside this, we found a main effect of dependent LE on mental health, irrespective of whether an individual experienced early institutional deprivation or not. It is important for caregivers, teachers, and other professionals to consider vulnerabilities to peer-related LE in adolescents with histories of institutional deprivation and work to redress the negative impacts of such exposure to protect them from further mental health problems in adulthood. Healthy schools and classrooms that welcome diversity and promote prosocial behavior can help protect the most vulnerable children and adolescents, and ultimately the mental health of the next generation of adults.

## Data Availability

Due to the disclosive nature of the data, it is not openly available. Upon request, anonymized, selected data may be made available. Requests to access these datasets should be directed to edmund.sonuga-barke@kcl.ac.uk.

## The ERA Young Adult Follow-Up Team

ES-B, MK, Jana Kreppner, Nicky Knights, Robert Kumsta, Barbara Maughan, and Wolff Schlotz.

## Appendix

**LE breakdown**

Peer problems (four items)

- Being rejected or seriously rebuffed by a friend
- People telling nasty stories about you
- Being physically bullied by other young people
- Being teased in a nasty way by other young people

Independent LE (17 items)

- Moving new home
- Birth of new brother or sister
- Changing to a new school
- Serious illness/death of a parent, brother or sister
- Parents separated/divorced
- Increased number of arguments between your parents
- Parent lost a job
- Brother/sister leaving home
- New stepmother/stepfather
- Death of a close friend
- adoption or fostering of new brother or sister
- major personal illness or injury
- serious illness or injury of a close friend
- being mugged, assaulted or beaten up outside school
- your home being burgled or broken into
- someone making unwelcome advances involving touching you
- hearing about death of birth parent

Dependent LE (six items)

- getting pregnant (getting girlfriend pregnant)
- Increase in number of arguments that you have with your parents
- getting into trouble with police
- breaking up boy-girlfriend
- being suspended/expelled from school
- giving birth (girlfriend giving birth)

## References

[1] Gunnar MR Bowen M. What was learned from studying the effects of early institutional deprivation. Pharmacology Biochem Behav. (2021) 210:173272–2. doi: 10.1016/j.pbb.2021.173272 PMC8501402 34509501

[2] Golm D Maughan B Barker ED Hill J Kennedy M Knights N. Why does early childhood deprivation increase the risk for depression and anxiety in adulthood? A developmental cascade model. J Child Psychol Psychiatry. (2020) 61:1043–53. doi: 10.1111/jcpp.13205 PMC8597399 32026473

[3] Kennedy M Kreppner J Knights N Kumsta R Maughan B Golm D. Early severe institutional deprivation is associated with a persistent variant of adult attention-deficit/hyperactivity disorder: clinical presentation, developmental continuities and life circumstances in the English and Romanian Adoptees study. J Child Psychol Psychiatry. (2016) 57:1113–25. doi: 10.1111/jcpp.2016.57.issue-10 PMC5042050 27264475

[4] Kennedy M Kreppner J Knights N Kumsta R Maughan B Golm D. Adult disinhibited social engagement in adoptees exposed to extreme institutional deprivation: Examination of its clinical status and functional impact. Br J Psychiatry. (2017) 211:289–95. doi: 10.1192/bjp.bp.117.200618 PMC5663971 28935662

[5] Pollak SD Nelson CA Schlaak MF Roeber BJ Wewerka SS Wiik KL. Neurodevelopmental effects of early deprivation in postinstitutionalized children. Child Dev. (2010) 81:224–36. doi: 10.1111/j.1467-8624.2009.01391.x PMC2846096 20331664

[6] Sonuga Barke EJ Kennedy M Kumsta R Knights N Golm D Rutter M. Child-to-adult neurodevelopmental and mental health trajectories after early life deprivation: The young adult follow-up of the longitudinal English and Romanian Adoptees study. Lancet. (2017) 389:1539–48. doi: 10.1016/S0140-6736(17)30045-4 28237264

[7] Wade M Fox NA Zeanah CH Nelson CA. Long-term effects of institutional rearing, foster care, and brain activity on memory and executive functioning. Proc Natl Acad Sci - PNAS. (2019) 116:1808–13. doi: 10.1073/pnas.1809145116 PMC6358707 30642973

[8] Wade M Carroll D Fox NA Zeanah CH Nelson CA. Associations between early psychosocial deprivation, cognitive and psychiatric morbidity, and risk-taking behavior in adolescence. J Clin Child Adolesc Psychol. (2022) 51:850–63. doi: 10.1080/15374416.2020.1864737 PMC8384982 33629920

[9] Rodriguez-Perez M Kennedy M Barker ED Kreppner J Solerdelcoll M Sonuga-Barke EJ. The adult outcome of childhood quasi-autism arising following extreme institutional deprivation. J Child Psychol Psychiatry. (2023) 64(9):1292–302. doi: 10.1111/jcpp.13767 36782398 PMC10476691

[10] Rutter M O'Connor TG. Are there biological programming effects for psychological development? Findings from a study of Romanian adoptees. Dev Psychol. (2004) 40:81. doi: 10.1037/0012-1649.40.1.81 14700466

[11] Bick J Zhu T Stamoulis C Fox NA Zeanah C Nelson CA. Effect of early institutionalization and foster care on long-term white matter development: A randomized clinical trial. JAMA Pediatr. (2015) 169:211–9. doi: 10.1001/jamapediatrics.2014.3212 PMC4413892 25622303

[12] Mackes NK Golm D Sarkar S Kumsta R Rutter M Fairchild G. Early childhood deprivation is associated with alterations in adult brain structure despite subsequent environmental enrichment. Proc Natl Acad Sci. (2020) 117:641–9. doi: 10.1073/pnas.1911264116 PMC6955353 31907309

[13] McLaughlin KA Sheridan MA Winter W Fox NA Zeanah CH Nelson CA. Widespread reductions in cortical thickness following severe early-life deprivation: A neurodevelopmental pathway to attention-Deficit/Hyperactivity disorder. Biol Psychiatry (1969). (2014) 76:629–38. doi: 10.1016/j.biopsych.2013.08.016 PMC3969891 24090797

[14] Sonuga-Barke EJ Schlotz W Kreppner J. V. Differentiating developmental trajectories for conduct, emotion, and peer problems following early deprivation. Monogr Soc Res Child Dev. (2010) 75:102–24. doi: 10.1111/j.1540-5834.2010.00552.x 20500635

[15] Kim KM. What makes adolescents psychologically distressed? Life events as risk factors for depression and suicide. Eur Child Adolesc Psychiatry. (2021) 30:359–67. doi: 10.1007/s00787-020-01520-9 32232580

[16] Song Y Li L Xu Y Pan G Tao F Ren L. Associations between screen time, negative life events, and emotional and behavioral problems among Chinese children and adolescents. J Affect Disord. (2020) 264:506–12. doi: 10.1016/j.jad.2019.11.082 31757618

[17] Lau JY Waters AM. Annual Research Review: An expanded account of information-processing mechanisms in risk for child and adolescent anxiety and depression. J Child Psychol Psychiatry. (2017) 58:387–407. doi: 10.1111/jcpp.2017.58.issue-4 27966780

[18] Assary E Vincent J Machlitt-Northen S Keers R Pluess M. The role of gene-environment interaction in mental health and susceptibility to the development of psychiatric disorders. In: Beyond Our Genes: Pathophysiology of Gene and Environment Interaction and Epigenetic Inheritance. Springer Nature (2020). p. 117–38. doi: 10.1007/978-3-030-35213-4_7

[19] Hammen C Henry R Daley SE. Depression and sensitization to stressors among young women as a function of childhood adversity. J Consulting Clin Psychol. (2000) 68:782–7. doi: 10.1037/0022-006X.68.5.782 11068964

[20] Bandoli G Campbell-Sills L Kessler RC Heeringa SG Nock MK Rosellini AJ. Childhood adversity, adult stress, and the risk of major depression or generalized anxiety disorder in US soldiers: A test of the stress sensitization hypothesis. psychol Med. (2017) 47:2379–92. doi: 10.1017/S0033291717001064 PMC5595661 28443533

[21] Espejo EP Hammen CL Connolly NP Brennan PA Najman JM Bor W. Stress sensitization and adolescent depressive severity as a function of childhood adversity: A link to anxiety disorders. J Abnormal Child Psychol. (2007) 35:287–99. doi: 10.1007/s10802-006-9090-3 17195949

[22] Kim AW Said Mohamed R Norris SA Richter LM Kuzawa CW. Psychological legacies of intergenerational trauma under south African apartheid: Prenatal stress predicts greater vulnerability to the psychological impacts of future stress exposure during late adolescence and early adulthood in soweto, South Africa. J Child Psychol Psychiatry. (2023) 64:110–24. doi: 10.1111/jcpp.13672 PMC10083984 35853622

[23] McLaughlin KA Conron KJ Koenen KC Gilman SE. Childhood adversity, adult stressful life events, and risk of past-year psychiatric disorder: a test of the stress sensitization hypothesis in a population-based sample of adults. psychol Med. (2010) 40:1647–58. doi: 10.1017/S0033291709992121 PMC2891275 20018126

[24] Wade M McLaughlin KA Buzzell GA Fox NA Zeanah CH Nelson CA. Family-based care buffers the stress sensitizing effect of early deprivation on executive functioning difficulties in adolescence. Child Dev. (2023) 94:e43–56. doi: 10.1111/cdev.13863 PMC9828738 36254858

[25] Jhang F. Uncontrollable and controllable negative LE and changes in mental health problems: Exploring the moderation effects of family support and self-efficacy in economically disadvantaged adolescents. Children Youth Serv Rev. (2020) 118:105417. doi: 10.1016/j.childyouth.2020.105417

[26] Technow JR Hazel NA Abela JRZ Hankin BL. Stress sensitivity interacts with depression history to predict depressive symptoms among youth: Prospective changes following first depression onset. J Abnormal Child Psychol. (2015) 43:489–501. doi: 10.1007/s10802-014-9922-5 PMC4423556 25123081

[27] Shapero BG Black SK Liu RT Klugman J Bender RE Abramson LY. Stressful LE and depression symptoms: The effect of childhood emotional abuse on stress reactivity. J Clin Psychol. (2014) 70:209–23. doi: 10.1002/jclp.22011 PMC3884028 23800893

[28] Gao Y Liang C Liu X Bai R Xing S. Self-esteem buffers the stress sensitizing effect of childhood maltreatment on adolescent nonsuicidal self-injury. J Affect Disord. (2024) 345:85–93. doi: 10.1016/j.jad.2023.10.117 37865345

[29] Wade M Zeanah CH Fox NA Tibu F Ciolan LE Nelson CA. Stress sensitization among severely neglected children and protection by social enrichment. Nat Commun. (2019) 10:5771–8. doi: 10.1038/s41467-019-13622-3 PMC6920417 31852902

[30] Rudolph KD Hammen C. Age and gender as determinants of stress exposure, generation, and reactions in youngsters: A transactional perspective. Child Dev. (1999) 70:660–77. doi: 10.1111/cdev.1999.70.issue-3 10368914

[31] Arseneault L. Annual Research Review: The persistent and pervasive impact of being bullied in childhood and adolescence: Implications for policy and practice. J Child Psychol Psychiatry. (2018) 59:405–21. doi: 10.1111/jcpp.12841 PMC6542665 29134659

[32] Rutter M Sonuga-Barke EJ Beckett C Castle J Kreppner J Kumsta R. Deprivation-specific psychological patterns: Effects of institutional deprivation. Monogr Soc Res Child Dev. (2010) 75(1):i–253. 10.1111/j.1540-5834.2010.00550.x 20500633

[33] Rizeq J Kennedy M Kreppner J Maughan B Sonuga-Barke E. Understanding the prospective associations between neuro-developmental problems, bullying victimization, and mental health: Lessons from a longitudinal study of institutional deprivation. Dev Psychopathol. (2022) 36:40–10. doi: 10.1017/S095457942200089X 35983788

[34] Crane C Heron J Gunnell D Lewis G Evans J Williams JMG. Adolescent over-general memory, life events and mental health outcomes: Findings from a UK cohort study. Memory. (2016) 24:348–63. doi: 10.1080/09658211.2015.1008014 PMC4743605 25716137

[35] Rutter M Bailey A Lord C. SCQ. The social communication questionnaire. Los Angeles: Western Psychological Services (2003).

[36] Goodman R. The Strengths and Difficulties Questionnaire: a research note. J Child Psychol Psychiatry. (1997) 38:581–6. doi: 10.1111/j.1469-7610.1997.tb01545.x 9255702

[37] American Psychiatric Association. Diagnostic and Statistical Manual of Mental Disorders. 5th edn. Arlington, VA: American Psychiatric Association (2013).

[38] Conners KC. Conners Comprehensive Behavior Rating Scales. Toronto, ON: MHS (2008).

[39] Starr LR Dienes K Stroud CB Shaw ZA Li YI Mlawer F. Childhood adversity moderates the influence of proximal episodic stress on the cortisol awakening response and depressive symptoms in adolescents. Dev Psychopathol. (2017) 29:1877–93. doi: 10.1017/S0954579417001468 29162191

[40] Fassett-Carman A Hankin BL Snyder HR. Appraisals of dependent stressor controllability and severity are associated with depression and anxiety symptoms in youth. Anxiety Stress Coping. (2019) 32:32–49. doi: 10.1080/10615806.2018.1532504 30303017 PMC6709974

[41] Park LE Crocker J. Contingencies of self-worth and responses to negative interpersonal feedback. Self Identity. (2008) 7:184–203. doi: 10.1080/15298860701398808

[42] Sowislo JF Orth U. Does low self-esteem predict depression and anxiety? A meta-analysis of longitudinal studies. psychol Bull. (2013) 139:213. doi: 10.1037/a0028931 22730921

[43] Moore SE Norman RE Suetani S Thomas HJ Sly PD Scott JG. Consequences of bullying victimization in childhood and adolescence: A systematic review and meta-analysis. World J Psychiatry. (2017) 7:60. doi: 10.5498/wjp.v7.i1.60 28401049 PMC5371173

[44] Bunea IM Szentágotai-Tătar A Miu AC. Early-life adversity and cortisol response to social stress: a meta-analysis. Trans Psychiatry. (2017) 7:1274. doi: 10.1038/s41398-017-0032-3 PMC5802499 29225338

[45] Thompson KN Agnew-Blais JC Allegrini AG Bryan BT Danese A Odgers CL. Do children with attention-Deficit/Hyperactivity disorder symptoms become socially isolated? longitudinal within-person associations in a nationally representative cohort. JAACAP Open. (2023) 1:12–23. doi: 10.1016/j.jaacop.2023.02.001 37312759 PMC10259183

[46] Bowlby J. Developmental psychiatry comes of age. America J Psychiatry. (1988) 145:1–10. doi: 10.1176/ajp.145.1.1 3276225

[47] Bowlby J. Attachment and loss: Attachment. New York: Basic Books (1982).

[48] O'Connor TG Bredenkamp D Rutter M English and Romanian Adoptees (ERA) Study Team. Attachment disturbances and disorders in children exposed to early severe deprivation. Infant Ment Health J. (1999) 20:10–29. doi: 10.1002/(SICI)1097-0355(199921)20:1<10::AID-IMHJ2>3.0.CO;2-S

[49] Sonuga-Barke E Kennedy M Golm D Knights N Kovshoff H Kreppner J. Adoptees’ responses to separation from, and reunion with, their adoptive parent at age 4 years is associated with long-term persistence of autism symptoms following early severe institutional deprivation. Dev Psychopathol. (2020) 32:631–40. doi: 10.1017/S0954579419000506 31190672

